# Prolapse of a Large Degenerating Uterine Leiomyoma: A Case Report

**DOI:** 10.7759/cureus.60622

**Published:** 2024-05-19

**Authors:** Austin B Wynn, Rachel L Shultz, Emily F Dayton, Kenneth Farmer

**Affiliations:** 1 Department of Research, Alabama College of Osteopathic Medicine, Dothan, USA; 2 Department of Obstetrics and Gynecology, Florida State University College of Medicine, Tallahassee, USA; 3 Department of Obstetrics and Gynecology, Southeast Health Medical Center, Dothan, USA

**Keywords:** prevent complications, uterine fibroid, quality care, pyomyoma, leiomyoma pathogenesis

## Abstract

Uterine leiomyomas (ULs) are common benign tumors seen in a large percent of women that can be classified based on their location within the uterus. They can cause a number of pelvic complications and can be managed medically, but more often surgically. Uterine pyomyomas often occur postpartum, possibly from infarction, and can lead to degeneration and sepsis. Our patient presents with a two-month development of a potential pyomyoma, found initially on computed tomography (CT). Office exam reveals a protruding mass from the cervical os, and removal was attempted but ultimately postponed for general anesthesia exam due to pain. The leiomyoma was removed and shown to be necrosing. Pyomyomas are often insidious and can often mimic other concerning pathologies. Modern imaging can show lesions within the pelvis but struggle to determine between fluid collection and possible infarcted masses. The importance of quality care measures in cases like this deserve to be emphasized to prevent serious complications.

## Introduction

Uterine leiomyomas (ULs) are common benign smooth muscle tumors found in the uterus of up to 70% of women of childbearing age [1]. ULs can be classified based on their location within the uterus. Types 1 and 2 are found within the submucous, type 3 contacts the endometrium, types 4-7 are subserosal, and type 8 is found outside the uterus [2,3]. While often asymptomatic or subclinical, up to 25% of women with ULs report a wide range of complications. Some common complications of UL are abnormal uterine bleeding, pelvic pain, lower urinary tract symptoms, and sexual dysfunction [4,5]. Less common complications of ULs are prolapse of fibroid and fibroid ischemia [6,7]. ULs are often managed surgically, although there are medical management options for those wishing to defer surgical treatment. These options include hormonal therapies such as gonadotropin-releasing hormone agonists or selective progesterone receptor modulators to decrease the size and volume of UL, nonsteroidal anti-inflammatory drugs (NSAIDs) to reduce blood loss, and tranexamic acid for severe blood loss [1]. 

Given that ULs are most commonly identified in women of childbearing age, there are some specific complications to be highlighted that may arise such as infertility, placental abruption, fetal malpresentation, preterm labor, and uterine pyomyoma [8]. A uterine pyomyoma often arises in the postpartum period likely secondary to infarction of the fibroid associated with blood loss during delivery, which then leads to degeneration and possible infection. We present a case of a woman two months postpartum with continued abdominal pain and abnormal bleeding due to a degenerating and prolapsed UL before any systemic signs of infection. Our report aims to aid in the prevalence of leiomyomas to prevent the development of postpartum complications and highlight the need for pelvic examination for patients presenting with pelvic pain. 

## Case presentation

**Figure 1:**
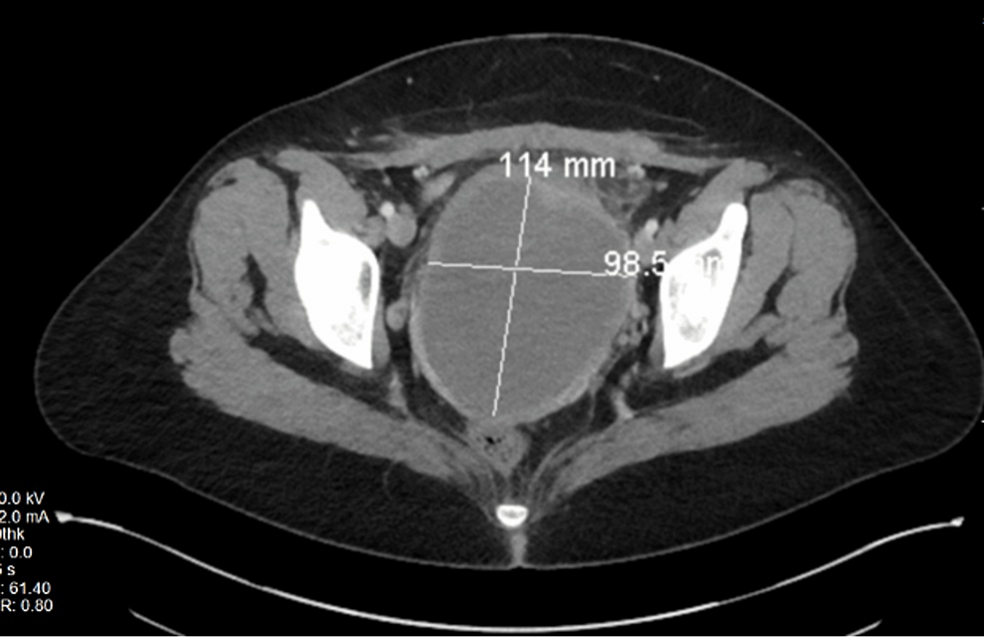
Pelvic CT showing potential mass in the pelvic cavity CT: Computed tomography Pelvic CT showing size of mass within the uterus measuring 11.4 cm x 9.85 cm. The mass takes up most of the space in the pelvic cavity, making the uterus difficult to see.

**Figure 2:**
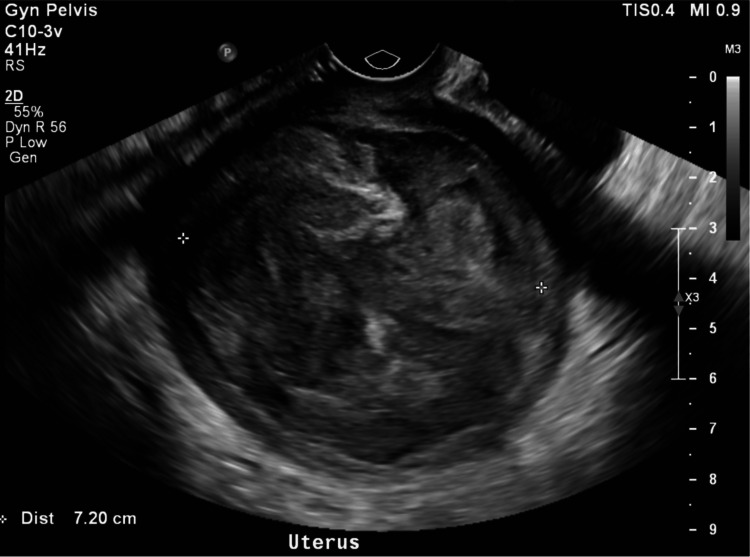
Transvaginal ultrasound of uterine mass A transvaginal ultrasound performed in office showed a well-defined, solid, concentric, hypoechoic mass in the uterus measuring 7.20 cm. The uterus is difficult to visualize due to the size and compression of the mass.

A 27-year-old Caucasian multiparous female presented with a two-month history of lower abdominal pain and vaginal bleeding. The patient stated that she has been having waxing and waning bleeding since her repeat low transverse cesarean section (C-section) was performed two months prior. During the C-section, a UL was found in the lower uterine segment and was left intact. The bleeding continued to get heavier with increased pain in the lower abdomen throughout the two months. The patient also noted a foul vaginal odor at postpartum presentation. The patient stated the odor and bleeding started a couple of weeks postpartum and slowly worsened over a few weeks. She had a computed tomography (CT) scan performed in the emergency room on two separate visits over a week, as seen in Figure 1. The scan was described as noting a large 11.4 cm x 9.85 cm x 9.85 cm hypodense complex collection or mass and uterine subserosal hematoma with an overall uterine size of 14.7 cm. A pelvic exam was not performed during the emergency department visits. We suspect that with a history of fibroids, imaging of the pelvis was the diagnostic preference in the emergency department. The patient was seen at the obstetrics office two weeks after her emergency department visits. A transvaginal ultrasound was performed which showed a 6.6 cm collection in the pelvis that did appear slightly smaller compared to the CT scan performed in the emergency department, as seen in Figure 2. The ovaries could not be visualized. A physical exam revealed that the Pfannenstiel incision had healed well, and no vulvar masses were present. There was no tenderness to the vaginal walls, erythema, or vaginal discharge. A pelvic exam revealed a beefy mass within the vagina that was protruding through the cervix. The mass was grasped with a ring forceps and an attempt to remove the mass was made. Due to patient discomfort, this was stopped in the office.

The patient was given cefazolin and transferred to the operating room for an exam under general anesthesia with a possible hysterectomy depending upon exam findings. She was intubated and placed under general anesthesia with no complications. During the exam, a degenerating mass was protruding from the dilated cervix. The mass was attached to the endometrium and removed from the uterus by blunt dissection and a ring forceps. The uterus was then washed with normal saline and inspected for further lesions. The mass was estimated at 8.0 cm x 8.0 cm and the source of the foul odor and bleeding. Pathology report indicated the mass to be an infarcted and degenerated leiomyoma. The patient was admitted for postoperative observation. Postoperative day one, the patient was ambulating well, tolerating a diet, and voiding without difficulty. Vaginal bleeding was minimal from the pedunculated source of the leiomyoma, and the foul odor was resolved immediately following removal of the mass. Pain was markedly improved, and she was discharged. A five-day course of levofloxacin 500 mg and metronidazole 500 mg was given.

## Discussion

Uterine pyomyomas are rare complications of ULs and may become life-threatening if not identified and treated early. The onset of further complications, such as necrosis, systemic infection, and septic shock, is often quite insidious and nonspecific as demonstrated in the presentation of this patient’s two-month history of abdominal pain following C-section [9]. Although this patient had not yet developed septic shock, her degenerating and necrotic fibroid would likely have progressed to shock and a fully developed pyomyoma if not treated promptly. There are very few cases of pyomyoma reported, less than 50 since 1945, and can often mimic other concerning pathologies such as tubo-ovarian abscess or pelvic inflammatory disease. However, the majority of the literature regarding uterine pyomyoma reports onset following uterine artery or fibroid embolization procedures, suggesting the role of infarction in the development of pyomyomas [10]. The importance of quality care measures in cases like this deserves to be emphasized here to prevent serious complications. The initial identification of the fibroid was a first-line quality measurement. The patient presented to the emergency department with pelvic pain. A pelvic exam was indicated and not performed, which could have identified the cause of pain weeks before the patient’s office visit. Fibroids should be identified and measured, along with patient presentation, to assess the need for intervention or watchful observation. Fibroids of varying sizes should be assessed for complications and location within the uterus to determine the need to remove them during C-section to prevent future complications.

## Conclusions

ULs, although common, may present in multiple locations within the uterus. The differing types of ULs can aid in determining the need for surgical intervention. ULs found in the lower uterine segment can cause increased complications via its location near the cervical os. This may warrant more aggressive management, especially in the perinatal period due to the risk of blood flow compromise. Although modern imaging can aid in assessing the risk of identified leiomyomas, this is not a replacement for a thorough physical exam. Pelvic exam can aid in the identification of protruding masses, as seen with our patient, along with vaginal wall defects, the presentation of pain, and assessment of internal pelvic structures. It is important to address concerns of pelvic pain before the development of pyomyomas and increased risk of sepsis.
